# Supplementary material for: Research priorities in children requiring elective surgery for conditions affecting the lower limbs: a James Lind Alliance Priority Setting Partnership
Source: BMJ Open. 2019 Dec 30;9(12):e033233. doi: 10.1136/bmjopen-2019-033233 (PMC6955494; doi:10.1136/bmjopen-2019-033233)
Supplement: Author's manuscript [file bmjopen-2019-033233.draft_revisions.pdf]

BMJ Open is committed to open peer review. As part of this commitment we make the peer review history of every article we publish publicly available.

When an article is published we post the peer reviewers' comments and the authors' responses online. We also post the versions of the paper that were used during peer review. These are the versions that the peer review comments apply to.

The versions of the paper that follow are the versions that were submitted during the peer review process. They are not the versions of record or the final published versions. They should not be cited or distributed as the published version of this manuscript.

BMJ Open is an open access journal and the full, final, typeset and author-corrected version of record of the manuscript is available on our site with no access controls, subscription charges or pay-per-view fees (http://bmjopen.bmj.com).

If you have any questions on BMJ Open's open peer review process please email info.bmjopen@bmj.com

BMJ Open

Research priorities in children requiring elective surgery for conditions affecting the lower limbs: a James Lind Alliance Priority Setting Partnership

Journal:	*BMJ Open*
Manuscript ID	bmjopen-2019-033233
Article Type:	Research
Date Submitted by the Author:	26-Jul-2019
Complete List of Authors:	Vella-Baldacchino, Martinique; Botnar Research Centre, Nuffield Department of Orthopaedics Rheumatology and Musculoskeletal Science<br>Perry, Daniel; Botnar Research Centre, Nuffield Department of Orthopaedics Rheumatology and MusculoskeletalScience<br>Roposch, Andreas; University College London Great Ormond Street Institute of Child Health Library<br>Nicolaou, Nicholas; Sheffield Children's NHS Foundation Trust<br>Cooke, Stephen; University Hospital Coventry<br>Ellis, Patricia; James Lind Alliance<br>Theologis, Tim; Oxford University Hospitals NHS Foundation Trust, Paediatric Orthopaedic Surgery
Keywords:	James Lind Alliance, lower limb surgery, research prioritisation

SCHOLARONE™
Manuscripts

Research priorities in children requiring elective surgery for conditions affecting the lower limbs: a James Lind Alliance Priority Setting Partnership

Vella-Baldacchino M, Perry DC, Roposch A, Nicolaou N and Cooke S, Ellis P, Theologis T

Martinique Vella-Baldacchino, Nuffield Department of Orthopaedics, Rheumatology and Musculoskeletal Science, Oxford, UK. (Martinique.Vella-Baldacchino@ouh.nhs.uk)

Daniel C Perry, Nuffield Department of Orthopaedics, Rheumatology and Musculoskeletal Science, Oxford, UK & Alder Hey Children's Hospital, Liverpool, UK. (danperry@doctors.org.uk)

Andreas Roposch, Institute of Child Health, University College London, London, UK. (a.roposch@ucl.ac.uk)

Nicholas Nicolaou, Sheffield Children's Hospital, Sheffield, UK. (nicolas.nicolaou@sch.nhs.uk)

Stephen Cooke, University Hospital of Coventry and Warwickshire, Coventry, UK. (stephen.cooke@uhcw.nhs.uk)

Patricia Ellis, James Lind Alliance Adviser. (jla@spencer.plus.com)

Tim Theologis, Nuffield Department of Orthopaedics, Rheumatology and Musculoskeletal Science, Oxford, UK. (timtheologis@gmail.com) - Corresponding Author.

Word count: 2,551 / 4,000

For peer review only

Abstract:

Objective: To identify and prioritise research questions concerning the elective surgical management of children with conditions affecting the lower limb by engaging patients, carers, and health care professionals.

Design: A modified nominal group technique.

Setting: UK

Participants: 388 individuals (29 patients, 155 parents/carers, 204 health care professionals) were recruited through hospital clinics, patient charities and professional organisations and participated in the initial prioritisation survey; 234 individuals took part in the interim prioritisation survey. 33 individuals (3 patients, 9 parents/carers, 11 health care professionals, 7 individuals representing the project's steering group and 3 James Lind Alliance facilitators) attended the final face-to-face workshop to rank the top 10 research priorities.

Interventions: Surveys were distributed using various media resources, such as newsletters, internet messaging boards and the 'Paediatric Lower Limb Surgery Priority Setting Partnership' website. Printed copies of the questionnaire were also made available to families in outpatient clinics.

Outcome measures: Survey results, top 10 and top 26 priority rankings

Results: The process took eighteen months to complete (July 2017-January 2019); 388 people generated 1023 questions; a total of 801 research questions were classified as true uncertainties. Following the James Lind Alliance methodology, 75 uncertainties were developed from the initial 801 questions. Twenty-six of those were selected through a second survey and were taken to the final face-to-face workshop where the top 10 research priorities were selected. The top-10 priorities included questions on Cerebral Palsy, common hip conditions (i.e. Perthes' Disease and Developmental Dysplasia of the Hip) as well as rehabilitation techniques and methods to improve shared decision making between clinicians and patients/families

Conclusions:

This is the first James Lind Alliance Priority Setting Partnership in children's orthopaedic surgery, a particularly under-researched and under-funded area. We have identified important research topics, which will guide researchers and funders to direct their efforts in future research.

For peer review only

ARTICLE SUMMARY

Strengths and Limitations of the Study

1. This was the first national research prioritisation exercise to identify research priorities amongst children affected by lower limb conditions.
2. There was balanced representation of patients, parents/carers and health care professionals.
3. There was a broad range of questions representing a variety of conditions and treatments.
4. Important questions applicable to the whole range of paediatric lower limb conditions were identified.
5. As a result of the wide variety of conditions included in this PSP, rare conditions did not make it to the top 10-research priority list, despite the evident research gaps.

INTRODUCTION

Musculoskeletal symptoms are the primary reason for referral to paediatric outpatient clinics: each year, one in eight children visits the doctor for musculoskeletal conditions, some of which are responsible for long-term impairment and disability[1]. Whilst musculoskeletal symptoms are common, the evidence underpinning their management is poor. In particular, orthopaedic surgical practice in children is almost exclusively based on poor-quality evidence[2-5]. This poor quality evidence has led to significant variation in surgical practices nationally and internationally[2-5]. This variation has resulted in conflicting information and loss of confidence in treatment pathways and sometimes in the clinicians that deliver them.

Clinical research may not truly represent the perceptions of clinicians' or patients' relating to which research questions are most important[6]. In 2003, Ian Chalmers published an idea in the Lancet promoting engagement, communication and discussion between researchers, patients, the public, carers and clinicians to agree on which treatment uncertainties mattered most to them and thus set formal future research priorities[6]. This became known as the James Lind Alliance (JLA), which has

For peer review only

since then evolved and spread[7]. This has given the opportunity to patients and members of the public to have an equal voice to clinicians and researchers in influencing the research agenda.

The JLA's infrastructure is funded by the National Institute for Health Research (NIHR) and oversees the overall process in a transparent and structured manner[8]. The NIHR encourages active involvement of the pubic and supports the JLA in feeding back research priorities to national funding bodies. There have been over 60 JLA projects investigating a range of treatment uncertainties, working with patients, carers and healthcare professionals and focusing on various clinical topics such as scoliosis, spinal cord injury, surgery for common shoulder problems and joint replacements for osteoarthritis [9].

The aim of the Paediatric Lower Limb Surgery (PLLS) Priority Setting Partnership (PSP) was to identify the unanswered questions on elective surgery for lower limb conditions in the paediatric population and agree by consensus on research priorities by forming a partnership between patients, parents/carers and clinicians.

METHOD

We followed the modified nominal group technique outlined in the step-by-step guidelines for the conduct of a James Lind Alliance Priority Setting Partnership. This technique has tested methods for PSPs to work effectively and reach credible and useful outcomes. In order for the JLA to fully endorse the final top-10 research priorities, the founding principles of patient and clinician involvement, transparency and systematic rigour had to be respected[10]. A JLA adviser (PE) was appointed by the National institute for Health Research (NIHR) Evaluation, Trials and Studies coordinating Centre (NETSCC) to support and guide the PSP setting process and liaise with the clinical lead. A Steering Group was appointed by the PSP lead, which consisted of charity representatives (LGW, EM), parents (HGO,CG), patients (DD), physiotherapists (CD, CB), allied health professionals (CW, EW), paediatric orthopaedic surgeons (DP, RA, NN, SC), a surgical trainee (MVB), the JLA advisor (PE) and the JLA PSP administrator (CR). JLA Paediatric Lower Limb Surgery PSP meetings were organised between July 2017 – January 2019.

Ethics

For peer review only - http://bmjopen.bmj.com/site/about/guidelines.xhtml

For peer review only

Consideration was given to applying for ethical approval for this study. Most PSPs do not require ethical approval, as no personal identifiable data are stored (http://www.jla.nihr.ac.uk/jla-guidebook/chapter-5/consent-and-ethics.htm). We have tested our study against the Health Research Authority - HRA criteria and were advised to proceed without an application for ethics approval.

Partner organisations: Identification and invitation

Partner organisations were identified through a process of peer knowledge and through the steering group members' networks. The organisations were invited to participate via a communication package describing the JLA Paediatric Lower Limb Surgery PSP objectives and process. Partners were firstly asked to provide their views and feedback on the proposed protocol of the partnership.

Organisations represented paediatric orthopaedic patients and their families or carers as well as relevant healthcare professionals, including medical doctors, nurses, physiotherapists and other allied health professionals with clinical experience of paediatric surgery for conditions affecting the lower limbs. Children and young people under the age of 16 years were encouraged to voice their views separately to those of their parents. Parents were asked to encourage their children to fill in a separate survey form.

Identifying treatment uncertainties

An online survey, agreed by the Steering Group, was set up and the link distributed to partner organisations. These organisations were encouraged to freely distribute the survey link and solicit research questions and uncertainties from their members. The steering group further encouraged the submission of questions from a broad array of individuals from across society using a variety of media, including newsletters, internet message boards and postal questionnaires. The link for the survey was available through the PLLS PSP website. Where printed copies of the questionnaire were made available, the data was entered into the online survey. The Steering group monitored responses to the survey and under-represented groups were targeted whilst the survey was live (January 2018 – March 2018).

Refining questions and uncertainties

The raw questions collected during the initial survey were organised into broad lower limb condition categories. Questions, which did not relate to the JLA PLLS objectives were excluded and labeled as 'Out of Scope'. The in-scope raw questions were further grouped under general indicative questions. The in-scope questions were then

For peer review only

searched using evidence published by the National Institute for Health and Care Excellence (NICE), Cochrane Library, systematic reviews and randomised control trials (Levels I and II). Evidence was only included if published in the English language over the past 10 years. A question was confirmed as a genuine uncertainty if it could not be answered using the literature search method above. Several topic experts in various fields were consulted to finalise decisions.

In-scope questions, which could be resolved with reference to existing literature, the 'unrecognised knowns', were identified and listed. This was then passed on to relevant partners within the steering group in order to communicate the information to the appropriate patient groups. By merging and grouping the remaining true uncertainties on similar themes together, the final number of research uncertainties was reduced to 75. These 75 questions were agreed by the Steering Group through a consensus process and were entered into the next stage of prioritisation. A list of the 75 questions can be found at http://www.jla.nihr.ac.uk/priority-setting-partnerships/Paediatric-lower-limb-surgery/.

Prioritisation – interim stage

The long list of 75 questions was reduced to a shorter list by a further online survey of the same partners, whereby respondents (a mixture of patients, parents and health care professionals) were asked to identify the 10 most important questions. Participants were asked to submit their preferences between 22nd August 2018 – 29th September 2018. The Steering Group reviewed the results of the second survey and agreed the top 26 questions to be taken to the final prioritisation meeting. This followed the James Lind Alliance recommendation to select approximately 25 questions for the final prioritisation. Care was taken to adequately represent the top choices of children, parents/carers and professional groups.

Prioritisation – final prioritisation workshop

The top 26 questions, which were most frequently selected in the interim prioritisation stage, were taken to the final face-to-face workshop. Thirty individuals, representing all relevant partners, were invited and asked to declare any conflicts of interests a priori. Care was taken to respect participants' views and ensure confidentiality of each individual's responses.

Discussions were led by three independent JLA facilitators, who had no previous experience in paediatric lower limb surgery. The established James Lind Alliance process was followed to reach consensus [10].

For peer review only

RESULTS

Figure 1 outlines the stages and process of the JLA PLLS PSP. The initial survey generated a total of 1023 questions from 388 individuals, consisting of a mixture of patients, carers, nurse educators, advanced nurse practitioners, clinical or healthcare scientists and clinicians (paediatricians, physicians, physiotherapists, occupational therapists, nurses, general practitioners and orthopaedic surgeons). A total of 47% of these questions were submitted by patients, parents or carers.

From the initial questions, 222 were marked as out of scope, as they did not adhere to the objectives of the JLA PLLS PSP (e.g. questions relating to spine or upper limb conditions). A total of 801 uncertainties were classified as in-scope by the steering group. The in-scope uncertainties were grouped into a preliminary list of 75 broader research questions. These were all confirmed as true uncertainties following a literature search, based on the JLA criteria and through consultation with topic experts.

The 75 research questions crystalised through this process were then prioritised through the interim survey, involving parents/carers, patients and health care professionals. A total of 234 individuals participated in the interim survey and selected 10 questions each, which they valued to be the most important uncertainties. From these participants, 117 (50%) were patients or parents, whilst the remaining were health care professionals.

Based on the interim prioritisation the Steering Group produced a shortlist of 26 uncertainties that were taken forward to the final face-to–face prioritisation workshop held in Oxford on 17 November 2018. A total of 30 individuals attended this workshop consisting of 6 physiotherapists, 9 parents, 4 patients, 7 orthopaedic surgeons, 1 clinical scientist, 1 advanced nurse practitioner and 2 charity representatives. Discussions were facilitated by three independent JLA facilitators and the top-10 unanswered research questions were agreed by all stakeholders present (Box 1).

Publicity

The steering group agreed on a dissemination strategy on 25 January 2019, using a variety of media such as an infographic, project report, conference presentations, online social media and the publication of a scientific paper.

Data Statement

For peer review only

All data relevant to this project are stored by the James Lind Alliance and are available on http://www.jla.nihr.ac.uk/priority-setting-partnerships/Paediatric-lower-limb-surgery/.

DISCUSSION

The JLA developed a transparent method to allow patients, carers and health care professionals to come together and establish a research agenda. Questions posed by patients or carers are given the same weighted importance to those submitted by health care professionals or academic scientists. Patient and public involvement is now recognised as best practice and is an essential requirement for research funding allocation by funders in the UK, Australia and United States of America [11].

This JLA process has identified the top-10 unanswered research priorities for surgery amongst children with conditions affecting the lower limbs. The questions identified through this JLA PSP require high quality research that will adequately address these uncertainties. The notable engagement of professionals, patients and the public, will ensure that the questions have a broad-reach in terms of real-world impact.

The number one priority was to identify the best way to measure outcomes following lower limb paediatric orthopaedic surgery. This highlights the importance of developing robust tools to be used in research to process and make informed decisions about clinical effectiveness.

Four of the top-10 priorities were directly related to the management of children with Cerebral Palsy. This is not surprising, as children with cerebral palsy often undergo interventions for the lower limbs and form a large part of paediatric orthopaedic surgical practice. The common hip conditions of childhood (Perthes' disease and Developmental Dysplasia of the Hip), as well as rehabilitation techniques and methods to improve shared decision making between clinicians and patients/families, also contributed to the top 10 list.

The priorities identified through this project are different to those identified through a Delphi process involving paediatric orthopaedic surgeons[12]. Whilst surgeons' questions focused around the management of specific conditions, the JLA PSP top

priorities also included generic questions on outcome measures, rehabilitation and access/communication. These differences highlight the importance of involving all relevant stakeholders, including those affected by the condition(s), when considering research priorities.

For those considering organising a JLA PSP, data organisation and management is a pivotal determinant to ensure the smooth running and success of the PSP. Adequate data management allows the linking of the questions that are developed during the later stages of the process with the originally submitted uncertainties and the stakeholders groups who submitted them. This is a JLA requirement, which underpins clarity and transparency.

The PSP received over 1,000 questions with balanced representation from patients and parents. In both the initial and the interim prioritisation surveys, approximately 50% of submissions were from patients or parents, indicating that they engaged well with our JLA PSP. We are confident that the final top-10 research priorities are representative of broad stakeholder involvement from patients, professionals and the wider public.

High quality research in paediatric lower limb surgery is a challenge, as many of the fundamental elements required to deliver a high-quality clinical trial are unknown; i.e. disease frequency, agreed outcomes, surgeon equipoise, surgical variation[2-3]. However, the IDEAL (Idea, Development, Exploration, Assessment, Long term Follow-Up) collaborative network has outlined a five-stage approach for assessing surgical interventions. IDEAL has encouraged the use of alternative study designs and prospective cohorts when randomised control trials are not feasible[13]. The IDEAL framework will provide the basis from which the evidence synthesis for many of the questions posed by this PSP will commence.

In recent years, there has been a cultural shift amongst surgeons in the UK towards evidence-based practice and evidence synthesis. We hope that the clear direction offered by this JLA PSP will enable clinicians, funders, researchers and patients to unite and urgently deliver robust answers to the questions highlighted by this PSP.

For peer review only

Additional low quality studies will only add to the noise within the literature and, as such, it is now time for definitive research to occur.

Author Contributions

TT, PE, DP, RA, NN, SC, MVB each made substantial contributions to this work, including study design, data collection, analysis and interpretation.

MVB drafted the initial manuscript and all authors (TT, PE, DP, RA, NN, SC) were involved in revising the manuscript and gave final approval of the version to be published. All authors had access to all data in the study and can take responsibility for the integrity of the data and the accuracy of the data analysis.

Funding

This study and process was funded by the British Society of Children's Orthopaedic Surgery (BSCOS), the British Orthopaedic Association (BOA) and the Oxford Biomedical Research Centre

Competing interests

None declared

Acknowledgements: The authors are grateful to a large number of people including, all steering group members: Camille Rougelot, Craig Walsh, Christine Douglas, Catherine Barry, Elizabeth Wright, Emma Morley, Loredana Guetg-Wyatt, Bert Martin, Helen Gregory-Osborn, Daniel Dolley, Catherine Ann Greaves . The authors would also like to thank all the participants who took all the patients and carers for contributing their questions and/or taking part in the interim survey and final workshop, all members of professional or charity organisations who contributed questions and invited patients to contribute, NETSCC for supporting and approving this PSP, BSCOS, BOA and the Oxford Biomedical Research Unit for funding the PSP.

The main partner organisations: British Society of Children's Orthopaedic Surgery (BSCOS), the British Orthopaedic Association (BOA), Oxford Biomedical Research Centre and STEPS Charity provided the foundation for this work.

For peer review only

References

1. Gunz AC, Canizares M, Mackay C, et al. Magnitude of impact and healthcare use for musculoskeletal disorders in the paediatric population: a population-based study. *BMC Musculoskelet Disord*. 2012; 13(98): 1-7
2. Dulai SK, Slobogean BL, Beauchamp RD et al. A quality assessment of randomized clinical trials in pediatric orthopaedics. *J Paediatr Orthop.* 2007; 27(5): 573-81
3. Dodwell E, Dua S, Dulai S, et al. The Quality of Randomized Controlled Trials in Pediatric Orthopaedics. *J Paediatr Orthop*. 2015;35(5):536-545.
4. Viehweger E, Jouve J, Simeoni M. Outcome evaluation in pediatric orthopedics. *Orthop Traumatol Surg Res.* 2014;100(1):S113-S123.
5. Chess L, Gagnier J. Risk of bias of randomized controlled trials published in orthopaedic journals. *BMC Med Res Methodol.* 2013;13(1).
6. Chalmers I, Atkinson P, Fenton M, et al. Tackling treatment uncertainties together: the evolution of the James Lind Initiative, 2003-2013. *JRSM* 2013; 106(12): 482-491
7. Partridge N, Scadding J. The James Lind Alliance: patients and clinicians should jointly identify their priorities for clinical trials. *Lancet* 2004; 364(9449): 1923-4
8. James Lind Alliance. Using the JLA logo. [Internet];Southampton: James Lind Alliance; 2018 [cited 2018 Sep 18]. Available from: http://www.jla.nihr.ac.uk/about-the-james-lind-alliance/using-the-jla-logo.htm
9. Lauren R, Stocks SJ, Alam R et al. Identifying primary care patient safety research priorities in the UK: a James Lind Alliance Priority Setting Partnership. BMJ Open. 2018;8:e020870. doi:10.1136/bmjopen-2017-020870
10. The James Lind Alliance Guidebook. 2018. http://www.jla.nihr.ac.uk/jla-guidebook/downloads/Print-JLA-guidebook-version-7-March-2018.pdf
11. Garry-Burrows, Willis T, Foy R et al. Role of patient and public involvement in implementation research: a consensus study. *BMJ Qual Saf.* 2018;0: 1-7
12. Perry DC, Wright JG, Theologis T et al. A consensus exercise identifying priorities for research into clinical effectiveness among children’s orthopaedic surgeons in the United Kingdom. *Bone Joint J.* 2018; 100B:680-4

13. The IDEAL Collaboration Framework. [Internet]: Oxford: The IDEAL Collaboration; [cited 2019 Jan 19]. Available from: http://www.ideal-collaboration.net

For peer review only

**1**	What are the best ways to measure outcome following lower limb orthopaedic surgery in children?
**2**	What should children's rehabilitation following orthopaedic surgery to the lower limbs include, how long is it expected to last and how does it affect the result of treatment?
**3**	What is the short-term and long-term clinical and cost effectiveness of orthopaedic lower limb surgery for children with Cerebral Palsy who can walk (considering best timing and technique)?
**4**	What are the short term and long term outcomes of surgery compared to non-surgical care in the treatment of Perthes disease?
**5**	What is the role of pre-operative rehabilitation in children presenting with lower limb orthopaedic conditions?
**6**	What is the short-term and long-term clinical and cost effectiveness of Selective Dorsal Rhizotomy (SDR) in children with Cerebral Palsy who can walk?
**7**	Can surveillance and non-surgical treatment (physiotherapy, botulinum toxin injections, functional electrical stimulation, orthotics, casting) prevent the development of deformity requiring surgery in children with Cerebral Palsy?
**8**	What is the best method of screening for Developmental Dysplasia of the Hip (DDH) in terms of clinical and cost effectiveness?
**9**	What are the best strategies to optimise communication of information between patients/carers and clinicians in order to enable shared decision making ?
**10**	What is the best management for hip displacement in children with Cerebral Palsy?

Box 1: Top 10 research priority areas from the James Lind Alliance Priority Setting Partnership for Paediatric Lower Limb Surgery Priority Setting Partnership

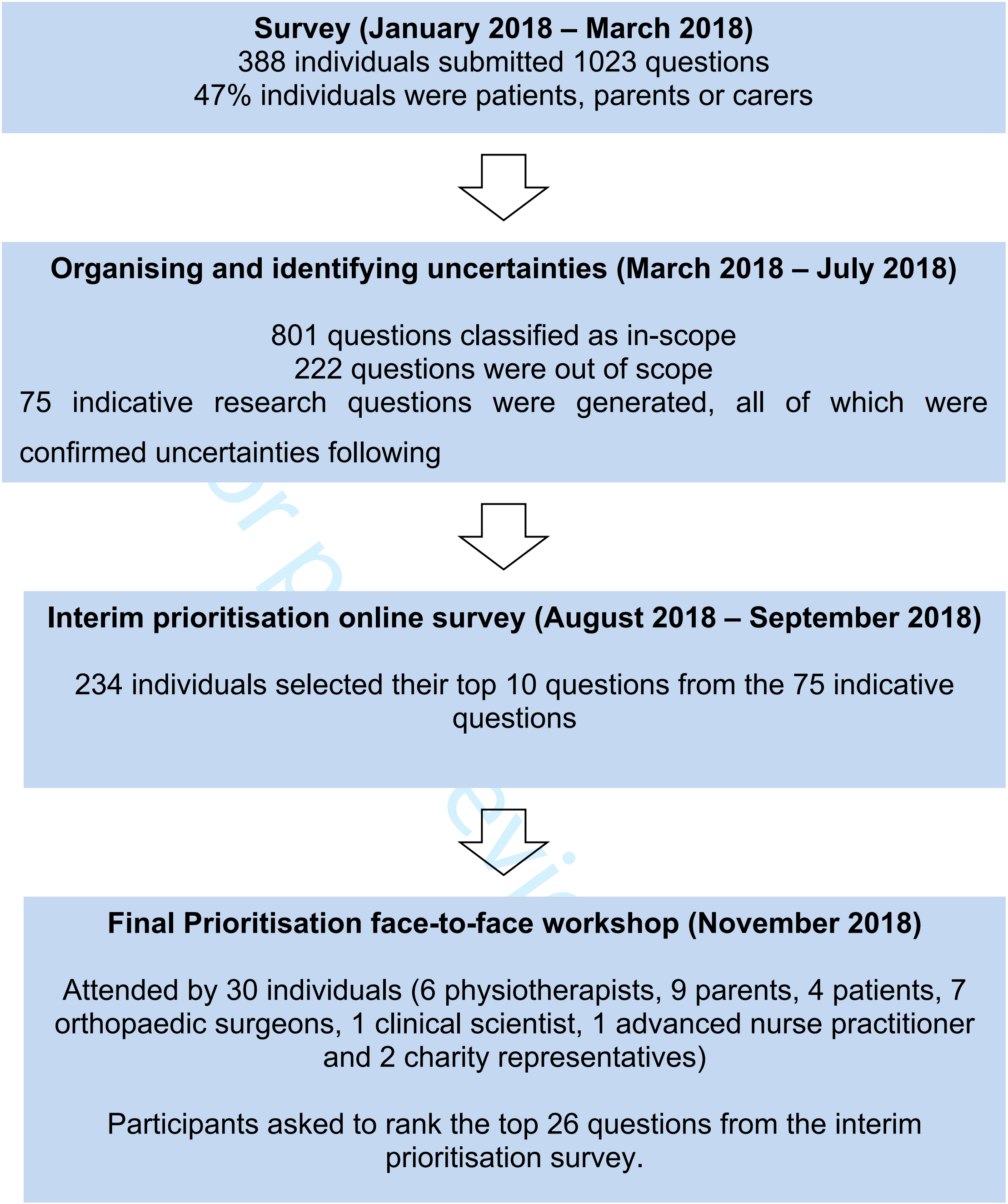

Figure 1: Summary of the James Lind Alliance Paediatric Lower Limb Surgery Priority Setting Partnership.

For peer review only - http://bmjopen.bmj.com/site/about/guidelines.xhtml

For peer review only

BMJ Open

Research priorities in children requiring elective surgery for conditions affecting the lower limbs: a James Lind Alliance Priority Setting Partnership

Journal:	*BMJ Open*
Manuscript ID	bmjopen-2019-033233.R1
Article Type:	Original research
Date Submitted by the Author:	12-Nov-2019
Complete List of Authors:	Vella-Baldacchino, Martinique; Botnar Research Centre, Nuffield Department of Orthopaedics Rheumatology and Musculoskeletal Science<br>Perry, Daniel; Botnar Research Centre, Nuffield Department of Orthopaedics Rheumatology and MusculoskeletalScience<br>Roposch, Andreas; University College London Great Ormond Street Institute of Child Health Library<br>Nicolaou, Nicholas; Sheffield Children's NHS Foundation Trust<br>Cooke, Stephen; University Hospital Coventry<br>Ellis, Patricia; James Lind Alliance<br>Theologis, Tim; Oxford University Hospitals NHS Foundation Trust, Paediatric Orthopaedic Surgery
**Primary Subject Heading**:	Surgery
Secondary Subject Heading:	Paediatrics
Keywords:	James Lind Alliance, lower limb surgery, research prioritisation

SCHOLARONE™
Manuscripts

Research priorities in children requiring elective surgery for conditions affecting the lower limbs: a James Lind Alliance Priority Setting Partnership

Vella-Baldacchino M, Perry DC, Roposch A, Nicolaou N and Cooke S, Ellis P, Theologis T

Martinique Vella-Baldacchino, Nuffield Department of Orthopaedics, Rheumatology and Musculoskeletal Science, Oxford, UK. (Martinique.Vella-Baldacchino@ouh.nhs.uk)

Daniel C Perry, Nuffield Department of Orthopaedics, Rheumatology and Musculoskeletal Science, Oxford, UK & Alder Hey Children's Hospital, Liverpool, UK. (danperry@doctors.org.uk)

Andreas Roposch, Institute of Child Health, University College London, London, UK. (a.roposch@ucl.ac.uk)

Nicholas Nicolaou, Sheffield Children's Hospital, Sheffield, UK. (nicolas.nicolaou@sch.nhs.uk)

Stephen Cooke, University Hospital of Coventry and Warwickshire, Coventry, UK. (stephen.cooke@uhcw.nhs.uk)

Patricia Ellis, James Lind Alliance Adviser. (jla@spencer.plus.com)

Tim Theologis, Nuffield Department of Orthopaedics, Rheumatology and Musculoskeletal Science, Oxford, UK. (timtheologis@gmail.com) - Corresponding Author.

Word count: 3,403 / 4,000

For peer review only

Abstract:

Objective: To identify and prioritise research questions concerning the elective surgical management of children with conditions affecting the lower limb by engaging patients, carers, and health care professionals.

Design: A modified nominal group technique.

Setting: UK

Participants: 388 individuals (29 patients, 155 parents/carers, 204 health care professionals) were recruited through hospital clinics, patient charities and professional organisations and participated in the initial prioritisation survey; 234 individuals took part in the interim prioritisation survey. 33 individuals (3 patients, 9 parents/carers, 11 health care professionals, 7 individuals representing the project's steering group and 3 James Lind Alliance facilitators) attended the final face-to-face workshop to rank the top 10 research priorities.

Interventions: Surveys were distributed using various media resources, such as newsletters, internet messaging boards and the 'Paediatric Lower Limb Surgery Priority Setting Partnership' website. Printed copies of the questionnaire were also made available to families in outpatient clinics.

Outcome measures: Survey results, top 10 and top 26 priority rankings

Results: The process took eighteen months to complete (July 2017-January 2019); 388 people generated 1023 questions; a total of 801 research questions were classified as true uncertainties. Following the James Lind Alliance methodology, 75 uncertainties were developed from the initial 801 questions. Twenty-six of those were selected through a second survey and were taken to the final face-to-face workshop where the top 10 research priorities were selected. The top-10 priorities included questions on Cerebral Palsy, common hip conditions (i.e. Perthes' Disease and Developmental Dysplasia of the Hip) as well as rehabilitation techniques and methods to improve shared decision making between clinicians and patients/families

Conclusions:

This is the first James Lind Alliance Priority Setting Partnership in children's orthopaedic surgery, a particularly under-researched and under-funded area. We have identified important research topics, which will guide researchers and funders to direct their efforts in future research.

For peer review only

ARTICLE SUMMARY

Strengths and Limitations of the Study

1. This was the first national research prioritisation exercise to identify research priorities amongst children affected by lower limb conditions.
2. There was balanced representation of patients, parents/carers and health care professionals.
3. There was a broad range of questions representing a variety of conditions and treatments.
4. Important questions applicable to the whole range of paediatric lower limb conditions were identified.
5. As a result of the wide variety of conditions included in this PSP, rare conditions did not make it to the top 10-research priority list, despite the evident research gaps.

INTRODUCTION

Musculoskeletal symptoms are the primary reason for referral to paediatric outpatient clinics: each year, one in eight children visits the doctor for musculoskeletal conditions, some of which are responsible for long-term impairment and disability[1]. Whilst musculoskeletal symptoms are common, the evidence underpinning their management is poor. In particular, orthopaedic surgical practice in children is almost exclusively based on poor-quality evidence[2-5]. This poor quality evidence has led to significant variation in surgical practices nationally and internationally[2-5]. This variation has resulted in conflicting information and loss of confidence in treatment pathways and sometimes in the clinicians that deliver them.

Clinical research may not truly represent the perceptions of clinicians' or patients' relating to which research questions are most important[6]. In 2003, Ian Chalmers published an idea in the Lancet promoting engagement, communication and discussion between researchers, patients, the public, carers and clinicians to agree on which treatment uncertainties mattered most to them and thus set formal future research priorities[6]. This became known as the James Lind Alliance (JLA), which has

For peer review only

since then evolved and spread[7]. This has given the opportunity to patients and members of the public to have an equal voice to clinicians and researchers in influencing the research agenda.

The JLA's infrastructure is funded by the National Institute for Health Research (NIHR) and oversees the overall process in a transparent and structured manner[8]. The NIHR encourages active involvement of the public and supports the JLA in feeding back research priorities to national funding bodies. There have been over 60 JLA projects investigating a range of treatment uncertainties, working with patients, carers and healthcare professionals and focusing on various clinical topics such as scoliosis, spinal cord injury, surgery for common shoulder problems and joint replacements for osteoarthritis [9].

Lower limb pathology forms the bulk of the elective practice in children's orthopaedic diseases, and these diseases share similar functional limitations in mobility. The aim of the Paediatric Lower Limb Surgery (PLLS) Priority Setting Partnership (PSP) was to identify the unanswered questions on elective surgery for lower limb conditions in the paediatric population and agree by consensus on research priorities by forming a partnership between patients, parents/carers and clinicians.

METHOD

We followed the modified nominal group technique outlined in the step-by-step guidelines for the conduct of a James Lind Alliance Priority Setting Partnership. This technique has tested methods for PSPs to work effectively and reach credible and useful outcomes. In order for the JLA to fully endorse the final top-10 research priorities, the founding principles of patient and clinician involvement, transparency and systematic rigour had to be respected[10]. A JLA adviser (PE) was appointed by the National institute for Health Research (NIHR) Evaluation, Trials and Studies Coordinating Centre (NETSCC) to support and guide the PSP setting process and liaise with the clinical lead. A Steering Group was appointed by the PSP lead, which consisted of charity representatives (LGW, EM), parents (HGO,CG), patients (DD), physiotherapists (CD, CB), allied health professionals (CW, EW), paediatric orthopaedic surgeons (DP, RA, NN, SC), a surgical trainee (MVB), the JLA advisor

For peer review only

(PE) and the JLA PSP administrator (CR). JLA Paediatric Lower Limb Surgery PSP meetings were organised between July 2017 – January 2019.

Patient and Public Involvement

This project was supported closely by patients, parents and organisations that represent them. Indeed these organisations drove much of the study, as they recognise the huge variability in orthopaedic practice, and identify that this makes it difficult to offer advice as each surgeon seemingly treats the same disease differently. Patient representatives were appointed to the steering group and contributed to the design and the smooth running of the study. As with all JLA PSPs, involvement of patients, parents and carers continued throughout the project, facilitated by the team and the JLA advisor. Approximately half of the submitted research questions were from patients and the public. Dissemination was delivered by both professional and lay members of the steering group through a variety of media such as infographics, a project report, conference presentations and online social media.

Ethics

Consideration was given to applying for ethical approval for this study. Most PSPs do not require ethical approval, as no personal identifiable data are stored (http://www.jla.nihr.ac.uk/jla-guidebook/chapter-5/consent-and-ethics.htm). We have tested our study against the Health Research Authority - HRA criteria and were advised to proceed without an application for ethics approval.

Partner organisations: Identification and invitation

Partner organisations were identified through a process of peer knowledge and through the steering group members' networks. The organisations were invited to participate via a communication package describing the JLA Paediatric Lower Limb Surgery PSP objectives and process. Partners were firstly asked to provide their views and feedback on the proposed protocol of the partnership.

Organisations represented paediatric orthopaedic patients and their families or carers as well as relevant healthcare professionals, including medical doctors, nurses, physiotherapists and other allied health professionals with clinical experience of paediatric surgery for conditions affecting the lower limbs. Children and young people under the age of 16 years were encouraged to voice their views separately to those of their parents. Parents were asked to encourage their children to fill in a separate survey form.

Identifying treatment uncertainties

For peer review only

An online survey, agreed by the Steering Group, was set up and the link distributed to partner organisations. These organisations were encouraged to freely distribute the survey link and solicit research questions and uncertainties from their members. The steering group further encouraged the submission of questions from a broad array of individuals from across society using a variety of media, including newsletters, internet message boards and postal questionnaires. The link for the survey was available through the PLLS PSP website. Where printed copies of the questionnaire were made available, the data was entered into the online survey. The Steering group monitored responses to the survey and under-represented groups were targeted whilst the survey was live (January 2018 – March 2018).

Refining questions and uncertainties

The raw questions collected during the initial survey were organised into broad lower limb condition categories. Questions, which did not relate to the JLA PLLS objectives were excluded and labeled as 'Out of Scope'. The in-scope raw questions were further grouped under general indicative questions. The in-scope questions were then searched using evidence published by the National Institute for Health and Care Excellence (NICE), Cochrane Library, systematic reviews and randomised control trials (Levels I and II). Evidence was only included if published in the English language over the past 10 years. A question was confirmed as a genuine uncertainty if it could not be answered using the literature search method above. Several topic experts in various fields were consulted to finalise decisions. The full list of questions submitted through the initial survey can be found here http://www.jla.nihr.ac.uk/priority-setting-partnerships/Paediatric-lower-limb-surgery/downloads/Paediactric-Lower-Limb-Surgery-PSP-final-data-sheet.pdf.

In-scope questions, which could be resolved with reference to existing literature, the 'unrecognised knowns', were identified and listed. This was then passed on to relevant partners within the steering group in order to communicate the information to the appropriate patient groups. By merging and grouping the remaining true uncertainties on similar themes together, the final number of research uncertainties was reduced to 75. These 75 questions were agreed by the Steering Group through a consensus process and were entered into the next stage of prioritisation. A list of the 75 questions can be found at http://www.jla.nihr.ac.uk/priority-setting-partnerships/Paediatric-lower-limb-surgery/downloads/Paediactric-Lower-Limb-Surgery-PSP-final-data-sheet.pdf.

For peer review only

Prioritisation – interim stage

The long list of 75 questions was reduced to a shorter list by a further online survey of the same partners, whereby respondents (a mixture of patients, parents and health care professionals) were asked to identify the 10 most important questions. Participants were asked to submit their preferences between 22nd August 2018 – 29th September 2018. The Steering Group reviewed the results of the second survey and agreed the top 26 questions to be taken to the final prioritisation meeting. This followed the James Lind Alliance recommendation to select approximately 25 questions for the final prioritisation. Care was taken to adequately represent the top choices of children, parents/carers and professional groups.

Prioritisation – final prioritisation workshop

The top 26 questions, which were most frequently selected in the interim prioritisation stage, were taken to the final face-to-face workshop. Thirty individuals, representing all relevant partners, were invited and asked to declare any conflicts of interests a priori. Care was taken to respect participants' views and ensure confidentiality of each individual's responses.

Discussions were led by three independent JLA facilitators, who had no previous experience in paediatric lower limb surgery. The established James Lind Alliance process was followed to reach consensus [10].

RESULTS

Figure 1 outlines the stages and process of the JLA PLLS PSP. The initial survey generated a total of 1023 questions from 388 individuals, consisting of a mixture of patients, carers, nurse educators, advanced nurse practitioners, clinical or healthcare scientists and clinicians (paediatricians, physicians, physiotherapists, occupational therapists, nurses, general practitioners and orthopaedic surgeons). A total of 47% of these questions were submitted by patients, parents or carers.

From the initial questions, 222 were marked as out of scope, as they did not adhere to the objectives of the JLA PLLS PSP (e.g. questions relating to spine or upper limb conditions). A total of 801 uncertainties were classified as in-scope by the steering group. The in-scope uncertainties were grouped into a preliminary list of 75 broader research questions. These were all confirmed as true uncertainties following a literature search, based on the JLA criteria and through consultation with topic experts.

For peer review only

The 75 research questions crystalised through this process were then prioritised through the interim survey, involving parents/carers, patients and health care professionals. A total of 234 individuals participated in the interim survey and selected 10 questions each, which they valued to be the most important uncertainties. From these participants, 117 (50%) were patients or parents, whilst the remaining were health care professionals.

Based on the interim prioritisation the Steering Group produced a shortlist of 26 uncertainties that were taken forward to the final face-to–face prioritisation workshop held in Oxford on 17 November 2018. A total of 30 individuals attended this workshop consisting of 6 physiotherapists, 9 parents, 4 patients, 7 orthopaedic surgeons, 1 clinical scientist, 1 advanced nurse practitioner and 2 charity representatives. Discussions were facilitated by three independent JLA facilitators and the top-10 unanswered research questions were agreed by all stakeholders present (Box 1).

Box 1: The top 10 research questions

**1**	What are the best ways to measure outcome following lower limb orthopaedic surgery in children?
**2**	What should children's rehabilitation following orthopaedic surgery to the lower limbs include, how long is it expected to last and how does it affect the result of treatment?
**3**	What is the short-term and long-term clinical and cost effectiveness of orthopaedic lower limb surgery for children with Cerebral Palsy who can walk (considering best timing and technique)?
**4**	What are the short term and long term outcomes of surgery compared to non-surgical care in the treatment of Perthes disease?
**5**	What is the role of pre-operative rehabilitation in children presenting with lower limb orthopaedic conditions?
**6**	What is the short-term and long-term clinical and cost effectiveness of Selective Dorsal Rhizotomy (SDR) in children with Cerebral Palsy who can walk?
**7**	Can surveillance and non-surgical treatment (physiotherapy, botulinum toxin injections, functional electrical stimulation, orthotics, casting) prevent the development of deformity requiring surgery in children with Cerebral Palsy?

For peer review only

**8**	What is the best method of screening for Developmental Dysplasia of the Hip (DDH) in terms of clinical and cost effectiveness?
**9**	What are the best strategies to optimise communication of information between patients/carers and clinicians in order to enable shared decision making ?
**10**	What is the best management for hip displacement in children with Cerebral Palsy?

Publicity

The steering group agreed on a dissemination strategy on 25 January 2019, using a variety of media such as an infographic, project report, conference presentations, online social media and the publication of a scientific paper.

Data Statement

All data relevant to this project are stored by the James Lind Alliance and are available on http://www.jla.nihr.ac.uk/priority-setting-partnerships/Paediatric-lower-limb-surgery/.

DISCUSSION

The JLA developed a transparent method to allow patients, carers and health care professionals to come together and establish a research agenda. Questions posed by patients or carers are given the same weighted importance to those submitted by health care professionals or academic scientists. Patient and public involvement is now recognised as best practice and is an essential requirement for research funding allocation by funders in the UK, Australia and United States of America [11].

This JLA process has identified the top-10 unanswered research priorities for surgery amongst children with conditions affecting the lower limbs. The questions identified through this JLA PSP require high quality research that will adequately address these uncertainties. The notable engagement of professionals, patients and the public, will ensure that the questions have a broad-reach in terms of real-world impact.

The number one priority was to identify the best way to measure outcomes following lower limb paediatric orthopaedic surgery. This highlights the importance of

For peer review only

developing robust tools to be used in research to process and make informed decisions about clinical effectiveness.

Four of the top-10 priorities were directly related to the management of children with Cerebral Palsy. This is not surprising, as children with cerebral palsy often undergo interventions for the lower limbs and form a large part of paediatric orthopaedic surgical practice. The common hip conditions of childhood (Perthes' disease and Developmental Dysplasia of the Hip), as well as rehabilitation techniques and methods to improve shared decision making between clinicians and patients/families, also contributed to the top 10 list.

The priorities identified through this project are different to those identified through a Delphi process involving paediatric orthopaedic surgeons[12]. Whilst surgeons' questions focused around the management of specific conditions, the JLA PSP top priorities also included generic questions on outcome measures, rehabilitation and access/communication. These differences highlight the importance of involving all relevant stakeholders, including those affected by the condition(s), when considering research priorities.

For those considering organising a JLA PSP, data organisation and management is a pivotal determinant to ensure the smooth running and success of the PSP. Adequate data management allows the linking of the questions that are developed during the later stages of the process with the originally submitted uncertainties and the stakeholders groups who submitted them. This is a JLA requirement, which underpins clarity and transparency.

The PSP received over 1,000 questions with balanced representation from patients and parents. In both the initial and the interim prioritisation surveys, approximately 50% of submissions were from patients or parents, indicating that they engaged well with our JLA PSP. We are confident that the final top-10 research priorities are representative of broad stakeholder involvement from patients, professionals and the wider public.

For peer review only

Participants were chosen to widely represent parent/patient groups and stakeholders, and could only choose from a pre-prioritised list of 26 questions. They were required to submit an expression of interest in taking part and a declaration of any vested interest of opinion. Furthermore, the final prioritisation meeting was supervised by three independent James Lind Alliance (JLA) Advisers. The JLA Advisers' role is to support and guide the meeting as expert neutral facilitators, ensuring that the process is followed in a fair, transparent way, with equal input from patients, carers and clinicians and their representatives, and equal consideration and debate given to each question prioritised. A key role of the JLA Adviser is to encourage understanding of the differing perspectives of patients, carers and clinicians, and to ensure that all voices are heard.

High quality research in paediatric lower limb surgery is a challenge, as many of the fundamental elements required to deliver a high-quality clinical trial are unknown; i.e. disease frequency, agreed outcomes, surgeon equipoise, surgical variation[2-3]. However, the IDEAL (Idea, Development, Exploration, Assessment, Long term Follow-Up) collaborative network has outlined a five-stage approach for assessing surgical interventions. IDEAL has encouraged the use of alternative study designs and prospective cohorts when randomised control trials are not feasible[13]. The IDEAL framework will provide the basis from which the evidence synthesis for many of the questions posed by this PSP will commence.

In recent years, there has been a cultural shift amongst surgeons in the UK towards evidence-based practice and evidence synthesis. We hope that the clear direction offered by this JLA PSP will enable clinicians, funders, researchers and patients to unite and urgently deliver robust answers to the questions highlighted by this PSP. Additional low quality studies will only add to the noise within the literature and, as such, it is now time for definitive research to occur.

Author Contributions

TT, PE, DP, RA, NN, SC, MVB each made substantial contributions to this work, including study design, data collection, analysis and interpretation.

For peer review only

MVB drafted the initial manuscript and all authors (TT, PE, DP, RA, NN, SC) were involved in revising the manuscript and gave final approval of the version to be published. All authors had access to all data in the study and can take responsibility for the integrity of the data and the accuracy of the data analysis.

Funding

This study and process was funded by the British Society of Children's Orthopaedic Surgery (BSCOS), the British Orthopaedic Association (BOA) and the Oxford Biomedical Research Centre

Competing interests

None declared

Acknowledgements: The authors are grateful to a large number of people including, all steering group members: Camille Rougelot, Craig Walsh, Christine Douglas, Catherine Barry, Elizabeth Wright, Emma Morley, Loredana Guetg-Wyatt, Bert Martin, Helen Gregory-Osborn, Daniel Dolley, Catherine Ann Greaves . The authors would also like to thank all the patients and carers for contributing their questions and/or taking part in the interim survey and final workshop, all members of professional or charity organisations who contributed questions and invited patients to contribute, NETSCC for supporting and approving this PSP, BSCOS, BOA and the Oxford Biomedical Research Unit for funding the PSP.

The main partner organisations: British Society of Children's Orthopaedic Surgery (BSCOS), the British Orthopaedic Association (BOA), Oxford Biomedical Research Centre and STEPS Charity provided the foundation for this work.

Figure Captions

Figure 1: Summary of the James Lind Alliance Paediatric Lower Limb Surgery Priority Setting Partnership.

References

For peer review only

1. Gunz AC, Canizares M, Mackay C, et al. Magnitude of impact and healthcare use for musculoskeletal disorders in the paediatric population: a population-based study. *BMC Musculoskelet Disord*. 2012; 13(98): 1-7
2. Dulai SK, Slobogean BL, Beauchamp RD et al. A quality assessment of randomized clinical trials in pediatric orthopaedics. *J Paediatr Orthop*. 2007; 27(5): 573-81
3. Dodwell E, Dua S, Dulai S, et al. The Quality of Randomized Controlled Trials in Pediatric Orthopaedics. *J Paediatr Orthop*. 2015;35(5):536-545.
4. Viehweger E, Jouve J, Simeoni M. Outcome evaluation in pediatric orthopedics. *Orthop Traumatol Surg Res*. 2014;100(1):S113-S123.
5. Chess L, Gagnier J. Risk of bias of randomized controlled trials published in orthopaedic journals. *BMC Med Res Methodol*. 2013;13(1).
6. Chalmers I, Atkinson P, Fenton M, et al. Tackling treatment uncertainties together: the evolution of the James Lind Initiative, 2003-2013. *JRSM* 2013; 106(12): 482-491
7. Partridge N, Scadding J. The James Lind Alliance: patients and clinicians should jointly identify their priorities for clinical trials. *Lancet* 2004; 364(9449): 1923-4
8. James Lind Alliance. Using the JLA logo. [Internet];Southampton: James Lind Alliance; 2018 [cited 2018 Sep 18]. Available from: http://www.jla.nihr.ac.uk/about-the-james-lind-alliance/using-the-jla-logo.htm
9. Lauren R, Stocks SJ, Alam R et al. Identifying primary care patient safety research priorities in the UK: a James Lind Alliance Priority Setting Partnership. BMJ Open. 2018;8:e020870. doi:10.1136/bmjopen-2017-020870
10. The James Lind Alliance Guidebook. 2018. http://www.jla.nihr.ac.uk/jla-guidebook/downloads/Print-JLA-guidebook-version-7-March-2018.pdf
11. Garry-Burrows, Willis T, Foy R et al. Role of patient and public involvement in implementation research: a consensus study. *BMJ Qual Saf*. 2018;0: 1-7
12. Perry DC, Wright JG, Theologis T et al. A consensus exercise identifying priorities for research into clinical effectiveness among children's orthopaedic surgeons in the United Kingdom. *Bone Joint J*. 2018; 100B:680-4

13. The IDEAL Collaboration Framework. [Internet]: Oxford: The IDEAL Collaboration; [cited 2019 Jan 19]. Available from: http://www.ideal-collaboration.net

For peer review only

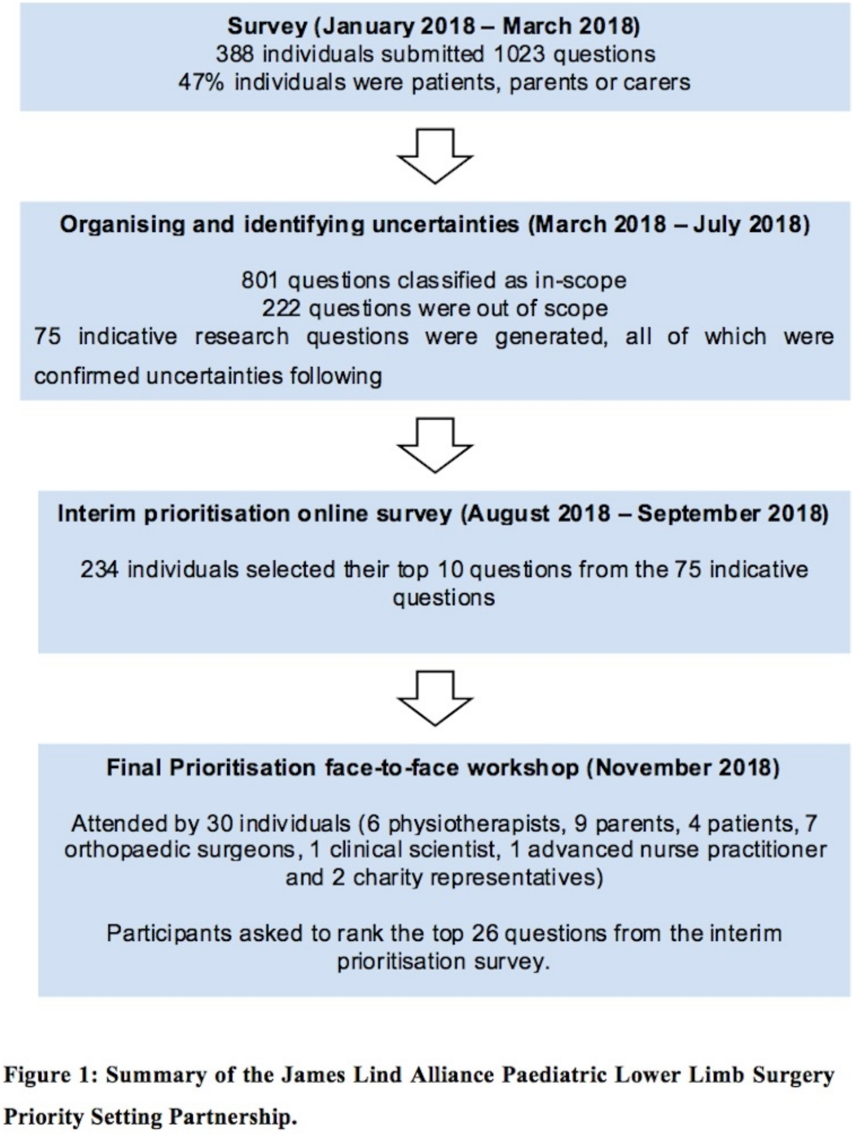

Figure 1: Summary of the James Lind Alliance Paediatric Lower Limb Surgery Priority Setting Partnership

70x89mm (300 x 300 DPI)
